# Deoxynivalenol: Toxicology, Degradation by Bacteria, and Phylogenetic Analysis

**DOI:** 10.3390/toxins14020090

**Published:** 2022-01-25

**Authors:** Anne Caroline Schoch Marques Pinto, Camilla Reginatto De Pierri, Alberto Gonçalves Evangelista, Ana Silvia de Lara Pires Batista Gomes, Fernando Bittencourt Luciano

**Affiliations:** 1 Graduate Program in Animal Science, School of Life Sciences, Pontifícia Universidade Católica do Paraná, 1155 Imaculada Conceição Street, Prado Velho, Curitiba 80215-901, Brazil; pinto.anne@pucpr.edu.br (A.C.S.M.P.); alberto.evangelista@pucpr.edu.br (A.G.E.); anaslpbg@gmail.com (A.S.d.L.P.B.G.); 2 Graduate Program in Sciences—Biochemistry, Department of Biochemistry and Molecular Biology, Federal University of Paraná, 100 Coronel Francisco H. dos Santos Avenue, Jardim das Américas, Curitiba 81530-000, Brazil; camillareginatto.p@gmail.com

**Keywords:** biodegradation, deoxynivalenol, phylogeny, DON, mycotoxins

## Abstract

Deoxynivalenol (DON) is a toxic secondary metabolite produced by fungi that contaminates many crops, mainly wheat, maize, and barley. It affects animal health, causing intestinal barrier impairment and immunostimulatory effect in low doses and emesis, reduction in feed conversion rate, and immunosuppression in high doses. As it is very hard to completely avoid DON’s production in the field, mitigatory methods have been developed. Biodegradation has become a promising method as new microorganisms are studied and new enzymatic routes are described. Understanding the common root of bacteria with DON degradation capability and the relationship with their place of isolation may bring insights for more effective ways to find DON-degrading microorganisms. The purpose of this review is to bring an overview of the occurrence, regulation, metabolism, and toxicology of DON as addressed in recent publications focusing on animal production, as well as to explore the enzymatic routes described for DON’s degradation by microorganisms and the phylogenetic relationship among them.

## 1. Introduction

Mycotoxins are toxic secondary metabolites produced by fungi that infect crops and can be produced in the field, during postharvest procedures, and in storage. The main genera involved in mycotoxin production are *Alternaria*, *Aspergillus*, *Cladosporium*, *Fusarium*, and *Penicillium* [1]. Around 400 mycotoxins have been described so far, and they differ in their structure, metabolization, and level of toxicological effects [2]. However, they are mostly stable to thermal processes and have negative effects on human and animal health [3].

Up to 80% of the grains produced worldwide are contaminated with at least one mycotoxin, but cooccurrence of two or more mycotoxins is very common, increasing their risk to human and animal health [4].

Among the mycotoxins characterized, trichothecenes are a group of sesquiterpenoids produced by *Fusarium* sp. that comprises deoxynivalenol (DON) and its acetylated forms, nivalenol (NIV), T-2 toxin, and HT-2 toxin [5]. In recent years, research on DON’s toxicology and mitigation methods has become more common because of DON’s high incidence across the world.

In this review, we aimed to bring an overview of occurrence, regulation, metabolism, and toxicology of deoxynivalenol as discussed in recent publications and explore the enzymatic routes currently described for DON’s degradation by microorganisms and the phylogenetic relationship among them, focusing on animal production. For this, we gathered studies that identified bacteria with the ability to transform DON into compounds that were less toxic than the parent toxin.

## 2. Results and Discussion

### 2.1. Occurrence, Regulation, Ingestion, and Metabolism

Grain contamination by mycotoxins may occur in the field or during storage, and factors such as temperature, high humidity, and handling are key points, as they can favor the production of mycotoxins [6]. 

DON is commonly found in temperate areas. Studies have reported that no DON has been identified in grains kept at water activity (a_w_) < 0.9 and temperatures lower than 11 °C [7]. Hope et al. [7] demonstrated that for both *Fusarium graminearum* and *F. culmorum*—main DON producers—ideal conditions for the toxin’s production are 25 °C and a_w_ > 0.98, which is in agreement with Ramirez and colleagues [8]. The use of fungicides can also stimulate DON’s production, especially in low doses, as ineffective doses promote mild to medium levels of stress to the fungus [7]. Among the practices of crop handling—e.g., fungicide and fertilizer application—the use of *Fusarium*-resistant cultivars seems to have the greatest positive influence on maintaining low levels of DON in grains. These cultivars were developed to present different genes that promote resistance to *Fusarium* head blight, a common wheat disease associated with DON [9]. 

DON, also known as vomitoxin, is the most prevalent mycotoxin according to the last survey report from Biomin [10], a referenced company in the field of mycotoxins, which analyzed 21,709 maize and wheat samples from all continents of the world. The study indicated high prevalence of DON in China (mainland and Taiwan), Middle East and Central America, where 86%, 78%, and 76% of the samples, respectively, were contaminated with the toxin. In South America, wheat samples presented an average of ≥1.5 µg/g of DON, representing a high risk for animal production, especially swine. In Oceania, this toxin was found in only 18% of samples. It is noteworthy that Oceania presented the lowest rate of all mycotoxins analyzed in this survey, and the risk for animal production was considered low to moderate. Higher concentrations of DON were observed in the 2020 survey than in the survey performed by Biomin in 2019, increasing the demand for more effective solutions for this issue.

Considering the panorama presented, regulations were established by governments enforcing maximum tolerable levels (MTL) of the toxin in foods and animal feed. Food intended for humans has its own regulation, which is more severe than that for animal feed, especially for foods destined for infants, who are more susceptible to the toxic effects of DON [11].

In 2004, the Food and Agriculture Organization (FAO) [12] released a worldwide survey on mycotoxin regulation status comparing the situations in 2003 and 1995. DON regulations were found in more countries after these nine years, although many of them considered these limits only for foods and not feed. In 2016, Romer Labs, a renowned company in the mycotoxin field, also released a survey showing that some countries are now regulating DON’s presence in feed [13]. Taken together, these data show a tendency toward more severe regulations, expanding DON control to other crops besides maize and wheat.

Legislation varies widely among the regulatory agencies of different countries. The European Commission (EC) has established detailed legislation, applying lower limits to swine production (0.9 µg/mL) than the Food and Drug Administration (FDA) of the United States (FDA), which recommends 5 µg/mL. South Africa and Canada established similar levels to those of the EC (1 µg/mL). Table 1 summarizes legislation established by the FDA [14]; the Canadian Food Inspection Agency [15]; the South African Department of Agriculture, Forestry, and Fisheries [16]; and the European Commission [17].

Some countries do not have official guidelines on MTL for animal feed. In Asia, each country makes its own regulations, and according to the Romer Labs report, China and Japan are the only countries that recommend MTL for DON in animal feed. Japan covers only specific groups, such as cows over 3 months of age [13]. Australia does not present specific regulations for this toxin in feed, probably because of the low levels found in the country and, therefore, the low incidence of mycotoxicosis in animals [18]. It is expected that in the coming years, with increased visibility of DON’s effects on the food chain supported by consistent research, new regulations will be established.

Deoxynivalenol MTLs were set only on cereals because DON presents a low occurrence in other products. Cereals are the main entry point for this mycotoxin in daily food and feed, because grains are a staple worldwide, especially maize and wheat. Swine and broilers are heavily exposed to DON, as their diets are composed mainly by grains, compared to other production animals [19].

Toxin daily intake is hard to measure in animals because of the different nutrient requirements of each species and even during different growth phases within the same species. Furthermore, as many countries do not have specific legislation for animal feed, and several types of feed can be used in this process, it is even harder to obtain a general estimate of the situation. What is certain is that both humans and animals are constantly exposed to DON, as it is not possible to totally extinguish fungal contamination in crops such as wheat and corn and thereby avoid the production of this toxin.

Once it is ingested by an animal, DON metabolization occurs in the intestine. In this process, some metabolites such as DON-3S, DON-GlcA, and DOM-1 may be generated in broilers [20] and in swine. The high transformation of DON into DON-3S and rapid elimination of the parent toxin may be the reasons why poultry is less susceptible to the effects of the toxin [20]. A suggested route for DON metabolism in poultry is its absorption in jejunum, transformation to DON sulfate forms (DON-3-S and DON-15-S) in the intestine, and excretion through bile and urine [21].

**Figure 1:**
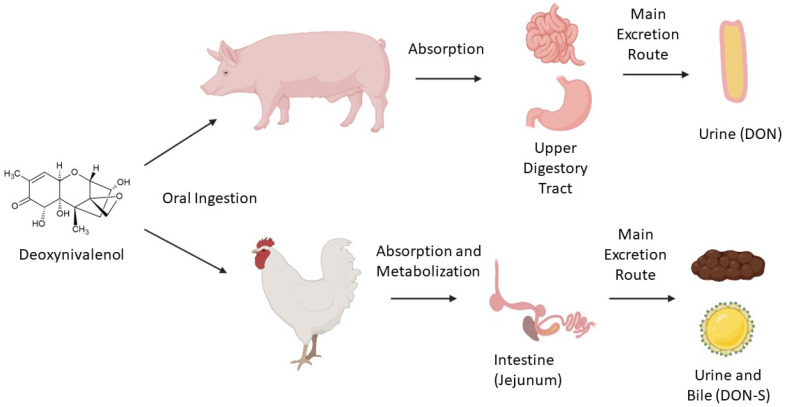
Main metabolization routes (simplified) of deoxynivalenol ingested orally by swine and poultry.

Swine are more sensitive to DON because of their high rate of absorption of the toxin in the upper digestive system, especially in the small intestine. Urine was found as the main excretion route, with DON being the best biomarker in this matrix, indicating a lower rate of metabolization by pigs than by poultry [22]. Microbiota composition is also a key factor for this toxin’s metabolization, as some microorganisms are capable of transforming the toxin into less toxic compounds, although this is not common in swine [23]. Fast tissue distribution was also observed, with 98% of metabolization occurring after 12 to 24 h; only traces were identified after this period [24]. Figure 1 summarizes poultry’s and swine’s main DON metabolization routes by oral ingestion.

Lack of research and adequate regulations for mycotoxins are a risk to animal feed safety worldwide. Regarding DON, most research has focused on swine, followed by poultry, and the metabolism in these organisms is well documented. In ruminants, recent studies demonstrating the metabolism of vomitoxin are scarce. Ruminants are considered resistant because of their robust microbiota, which can transform the toxin into less toxic metabolites [25]. However, evidence has pointed to modulatory effects of mycotoxins on the intestinal microbiota of ruminants, which must be further investigated [26].

Soon, more severe control of DON is expected, as MTLs for this toxin tend to be included in countries where it is not regulated. It should also be more severely controlled than the existent regulations, especially as climate change can favor its production [27].

### 2.2. DON’s Mechanisms of Toxicity

DON is classified as a sesquiterpenoid and possesses in its structure an epoxy group at C12–13 and hydroxyl groups at C3, C7, and C15, which are mainly responsible for its toxicity [28,29].

Recent studies have shown a change in gene regulation as one effect of DON exposure, mainly affecting immune response genes, especially those linked to cytokines, which are signaling molecules that regulate the inflammatory response. There has also been evidence of disruption in the expression of genes related to nutrient transport, barrier function, cell cycle regulation, and mitochondrial function, leading to malfunction of the animal cell [30,31]. In high doses (e.g., 8 µg/g of feed), DON can suppress genes related to immune response [32]. DON has also presented upregulation of apoptotic gene expression, leading to cell death of hippocampal nerve cells in piglets [33].

At a molecular level, DON affects ribosomal activity by binding into the 60S unit and inducing ribotoxic stress, leading to deficient protein synthesis. Changes in the mitochondrial structure and functioning were also observed. It also causes activation of mitogen-activated protein kinases (MAPK), leading to impairment of cell proliferation and apoptosis [33]. 

DON exerts its toxicity mainly in the gastrointestinal tract (GIT), and when in high doses, it provokes a reduction in goblet cell production. These cells are responsible for mucus production and help to maintain the integrity of the intestinal barrier. DON also affects the expression of tight junction proteins, such as claudins, that are responsible for regulating epithelial cell permeability and cell adhesion in the intestine [34]. This is especially worrying because the GIT enables adequate nutrient absorption, and this function may be impaired.

The intestinal barrier and a healthy microbiome also protect the animal against pathogens, and they are both negatively affected by DON [35]. Differences between the microbiota in the small intestines of weaning piglets fed with DON and those fed with a DON-free diet were reported [36]. Clear signals of dysbiosis, such as decrease in the population of Firmicutes—involved in the metabolism of nutrients and maintenance of intestinal health—and increased presence of Actinobacteria were noticed in piglets fed with DON. Similar results were found in weaning rabbits, with decreased microbiota diversity under the presence of high levels of DON [37].

Microbiota also play an important role in protecting the host from pathogen growth along with the immune system, which also suffers under the effects of DON. Changes in the T-cell differentiation pattern, decreasing the proliferation of cells that are directly involved in immune response, were found [38]. This result was supported by Cai and colleagues [39], who described a decrease in naïve cell differentiation into antibody-secreting cells due to lower cytokine receptor expression on the cell surface. Furthermore, they demonstrated that the toxin affected the immune response of mice infected with *Listeria monocytogenes*, intensifying the infection. 

Alterations in the reproductive cycle have also been noticed in animals intoxicated with DON. The mycotoxin (2 µg/mL) has provoked disruption in the hormonal cycle, stimulating the release of progesterone and estrogens in vitro in porcine ovarian granulosa cells [40]. Disruption of the histological pattern and impairment of follicular development in ovarian explants of pigs were also demonstrated [41].

The toxin also restrained testicular development causing anomalies in its structure and impaired blood–testis barrier integrity in mice [42]. Sperm viability was also decreased, and morphology alteration of the gametes was found, results that were supported by Tassis et al. [43] in their study with boar semen and Yang et al. [44] in their study with BALB/c mice. One study also indicated that testicular function was not the only factor negatively affecting the male reproductive function and that neuroendocrine activity may suffer important alterations as an effect of DON [42]. Altered activity in the brains of piglets was suggested, especially in the release of neurotransmitters responsible for physiological and nervous system regulations, such as decreases in dopamine and GABA and increases in norepinephrine and 5-hydroxytryptamine [45]. One possible effect is the modulation of appetite.

The brain cell morphology of piglets was also altered by DON, with a lack of organelle and vacuole formation when challenged with 2.2 µg/g of the toxin added to the feed. DON also decreased the antioxidant activity in the brain because of a reduction in superoxide dismutase and glutathione peroxidase activity [45]. Furthermore, an increase in blood–brain barrier permeability and a decrease in cell viability were found in in vitro models as well as in rats, chickens, pigs, and mice. All together, these studies point to brain activity disorders and homeostasis imbalance [46].

**Figure 2:**
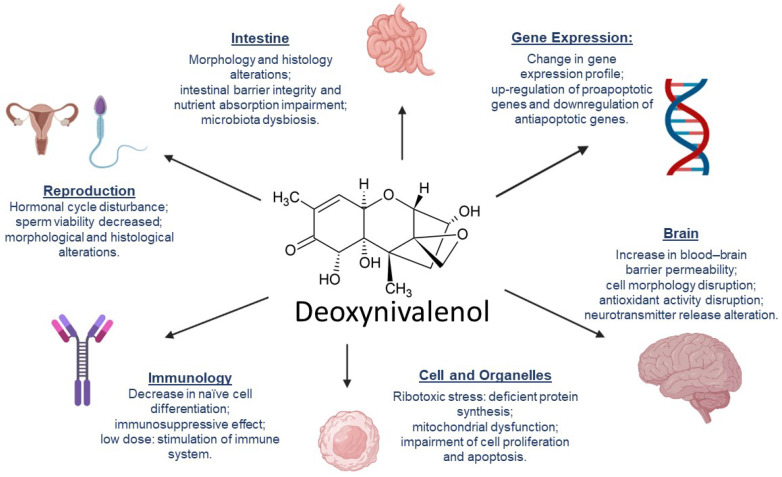
Effects of deoxynivalenol in animal organs and systems.

Other studies have reported that other organs, namely the liver [47], kidney, and spleen [48], are also affected by DON. This results in immunosuppression, metabolic alterations, and disturbance in the amino acid production profile, leading to malfunction of physiological processes. Figure 2 summarizes the effects of DON on targeted animal organs and systems.

DON’s toxicity in vivo is well documented. Negative impacts were reported in grass carp [49], broilers [50,51], piglets [36,52], finishing pigs [53], mice [54,55], and rabbits [56]. Among food animals, a predominance of studies involved pigs and piglets rather than broilers, probably because of the higher tolerance to the toxin presented by broilers. Important factors to be considered for the severity of the toxin’s effect are dose and time of exposure, which determine the outcome of the intoxication. Serviento and colleagues [53] compared three treatment groups of finishing pigs fed with 3 µg/g of DON. The first group was exposed to the toxin once a day from 113 to 119 d of life; the second group was exposed to DON once a day from 134 to 140 d; and the third group was exposed to the toxin in both periods. The results suggested that pigs’ tolerance to DON’s presence increased in the second exposure after 4 weeks, probably because of adaptations of their microbiota. They also showed that previous contact with the toxin did not avoid adverse effects of later exposures. However, it improved animal recovery from a second exposure to contaminated feed. In addition, it was observed that older animals exposed to the toxin presented lower average daily feed intake and daily weight gain than those challenged in early periods, indicating that age is also an important parameter to be considered.

Studies have shown immunosuppression in animals subjected to high doses of the toxin [29,37,38,56]. However, a recent study showed that low doses of DON can stimulate the immune system, increasing lymphocyte and goblet cell numbers and activating signaling pathways with an increased production of cytokines, suggesting a dose-dependent effect in the immune response [57]. Alassane-Kpembi et al. [58] pointed out that commonly used detection methods may fail to identify the potential harms from low-exposure doses of DON and that omics have the potential to provide specific fingerprints about the mycotoxin’s effects.

The masked forms of DON also represent a threat to food security, and they are often neglected. Acetyl and glycosylated modifications are among the most common masked forms, and some of them may be more toxic than DON itself. Studies have shown faster absorption of acetylated forms and toxic effects, such as activation of the MAPK signaling pathway, similar to those of DON. In addition, digestive enzymes and microorganisms can transform 15-A-DON and 3-A-DON in DON [59].

Because of the diverse toxic effects of the toxin in different systems of the animal organism, some strategies have been developed to mitigate them. Biodegradation is a potential mechanism to decrease DON’s toxicity and has been widely studied in recent years. The most reported biodegradation metabolites are DOM-1 and 3-epi-DON, which were found to be less toxic than the parent toxin [60,61]. Their characteristic lower toxicity was confirmed by Bracarense and colleagues [52] in vivo using piglets fed with 3 µg/g of DON, DOM-1, and 3-epi-DON. Results demonstrated histological modifications and proinflammatory response in the intestines, livers, and lymph nodes of animals treated with DON. However, those treated with DOM-1 and 3-epi-DON presented similar scores as those in the control group. 

Reduction in T-cell proliferation and disruption to the expression of molecules involved in immune response were observed in vitro when cells were exposed to 1.6 µM of DON, yet a 10-fold higher dose of DOM-1 did not exert any of these effects [62]. Mayer and colleagues [63], working with five different cell lines—mice macrophages (RAW 264.7), porcine intestinal cells (IPEC-1 and IPEC-J2), trout gill (RTgill-W1), and human liver cell (HepG2)—found similar results.

Toxic effects of DON are well documented, especially in swine, which has been found the most sensitive species to DON in animal production. Although many in vitro studies have reported a sharp drop in toxicity via microbial transformation, generating DOM-1 and 3-epi-DON, there is a lack of studies of in vivo toxicity, especially about 3-epi-DON, which was described in the literature later than DOM-1. 

In vivo analyses often require special evaluation and authorization from the ethics committee on the use of animals, an additional step that is often bureaucratic and time consuming, although necessary. Further in vivo studies are required to fully confirm the lower toxicity of these metabolites, as systemic effects cannot be fully evaluated in in vitro studies.

### 2.3. Biodegradation of Deoxynivalenol

Biodegradation is a process that consists of the degradation of one compound into another mediated by living organisms, such as bacteria, yeast, or fungi. In the mycotoxin context, it is interesting that the subproducts generated in this process are less toxic than the parent toxin and may not negatively affect either animal or plant cells.

For some mycotoxins, such as aflatoxin B1, the most common approach for mitigation in animal production is to include feed additives such as binders, which can reach 90+% adsorption of the toxin in the GIT of livestock animals [64]. However, DON has a low affinity to binders in general due to its structural low polarity. An in vitro study showed that bentonite clays, cellulose products, yeast cell wall products, and activated charcoal products had adsorption rates of 3.24%, 11.6%, 22.9%, and 14.4% of DON, respectively, which are not effective results when applied in animal feed [64]. Therefore, development products containing microorganisms with the ability to degrade DON into less toxic compounds represent an interesting strategy. Few works have discussed fungi and yeast such as *Aspergillus tubingensis* [65], *Aspergillus oryzae* and *Rhizopus oryzae* [66], and *Saccharomyces pastorianus* [67] as DON degraders. Most studies have isolated bacteria from different genera presenting a variety of degradation pathways. The first report of a DON-degrading microorganism was made by King and colleagues [68] in rumen fluid, with DOM-1 as a subproduct and no identification of the species involved in this process. The first organism to be described as a DON detoxifier was part of the *Agrobacterium*–*Rhizobium* group and called strain E3-39 [69]. Since then, many other species have described (Table 2), and other degradation subproducts, such as 3-keto-DON [59], 3-epi-DON [70], and 3-epi-DOM-1 [71], have been identified.

The first reports were made in anaerobic conditions, because ruminants showed less susceptibility to DON toxicity than monogastric animals [98]. Therefore, it was plausible to assume that rumen bacteria played an important role in DON metabolization. Many other sources were used to collect potential degrading microorganisms, including fish [99] and chicken intestine [77], environmental samples from water [89], soil [72], and plants [82]. In the last 10 years, sampling from soil or plants in aerobic conditions has become more common for screening because of the observation that although toxins are frequently found in grains, they are not found in the soil where these plants are cultivated. 

Many researchers have used the media enrichment method, which involves adding the toxin into culture media and proceeding with several subcultures, aiming to find potential DON degraders [69,75,81]. This technique favors the microorganisms that metabolize DON, especially when it is used as a single carbon source. In this case, DON degraders become predominant in the sample and can be easily isolated [97]. It is also possible, although less common, to test this ability with single species already isolated. This technique consists of cultivation of the microorganism in the presence of the toxin and evaluation of its degradation rate [100].

### 2.4. Enzymatic Pathways

Understanding the enzymatic pathways through which DON biodegradation occurs is crucial for the development of products containing microorganisms with such ability. Cofactors of the enzymatic reactions must be considered and identified as well [101]. Studies have shown different routes for enzymatic degradation of the toxin, mainly de-epoxidation and epimerization, including- extra and intracellular enzymes and aerobic and anaerobic pathways.

**Figure 3:**
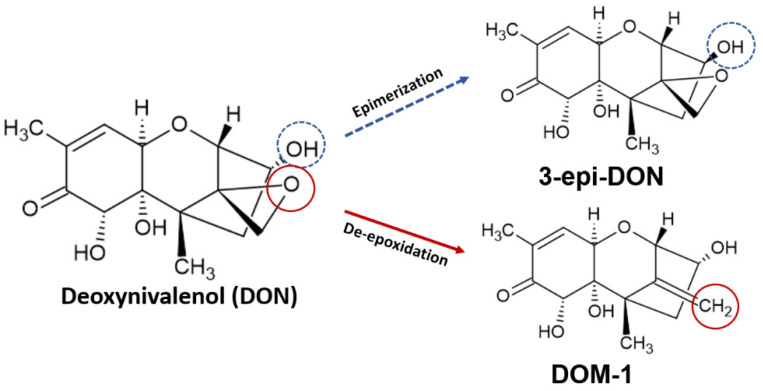
Epimerization and de-epoxidation of deoxynivalenol, producing 3-epi-deoxynivalenol (3-epi-DON) and deepoxydeoxynivalenol (DOM-1), respectively.

The de-epoxidation reaction of vomitoxin involves the removal of an oxygen atom and incorporation of three hydrogen atoms, forming DOM-1 [102]. Epimerization takes place when the -OH radical in the C3 carbon is epimerized through an isomerization reaction, forming 3-epi-DON [78]. Both reactions are demonstrated in Figure 3. The two metabolites generated in de-epoxidation and epimerization reactions of DON were well described in in vitro studies and were found to be less toxic than the parent toxin.

Degradation of DON by intracellular enzymes was described by Zhang et al. [89]. In this study, the cell lysate of *Pelagibacterium halotolerans*, isolated from sea water, showed the strongest DON-degrading ability (72.5% of 500 µg/mL) under 30 to 40 °C and pH 8 to 10. Acid pH (lower than 6) and heat (100 °C for 10 min) greatly decreased the degradation of the mycotoxin, leading to the conclusion that this degradation was mediated by pH-dependent enzymes.

Jia and colleagues [76] isolated the supernatant of *Bacillus subtilis* ASAG 216 grown in Luria–Bertani broth, which was subsequently incubated with 100 µg/mL of DON. A degradation rate of 81.8% was found at 35–50 °C and pH 6.5–9. Heat, sodium dodecyl sulfate, and proteinase K treatments significantly reduced DON’s degradation, which indicated that extracellular enzymes were part of this process.

Aerobic and anaerobic conditions also play an important role on DON’s epimerization by microorganisms [93]. A microbial consortium was isolated from agricultural soil with the capacity to totally degrade 50 µg/mL of DON at 27 °C in nutrient broth and mineral media enriched with 0.5% (*w*/*v*) peptone. In this case, aerobic degradation (60 h) was faster than anaerobic degradation (96 h), which may be explained by the composition of the consortium involving mostly aerobic or facultative anaerobic organisms. He et al. [78] also demonstrated a relationship among oxygen presence, population increase, and DON degradation in which aerobiosis increased cell proliferation and DON degradation.

In anaerobic cultivation, full transformation of DON to DOM-1 was achieved at 37 °C using a consortium of *Bacillus* spp., *Clostridiales*, *Anaerofilum* sp., and *Collinsella* sp. originated from chicken intestine [77]. Anaerobic conditions were also used in *Eubacterium* cultivation with the formation of the same subproduct, DOM-1 [73].

No study performing experiments in anaerobic conditions was found in which 3-epi-DON was formed as a by-product. Bacteria capable of degrading DON into 3-epi-DON were mainly from environmental sources such as wheat, soil, and water, so the production of this metabolite may be conditioned to the species that are prevalent in these environments. Furthermore, 3-epi-DON was reported in more studies than DOM-1, probably because of the aerobic sources used. Production of DOM-1 seems to be prevalent in anaerobic environments such as the intestine and rumen, since only a few studies have reported DOM-1 production under aerobic conditions.

Hassan et al. [103] worked with cell lysates of *Devosia mutans* in aerobic conditions and described a two-step epimerization of DON involving the oxidation of DON to 3-keto-DON and a reduction to 3-epi-DON with NADPH as an enzymatic cofactor. They also showed that the enzymes involved in the two reactions were physically separated. He et al. [83], also working with *Devosia* strain D6-9, found the same degradation pattern. 

A hydroxylation reaction was reported, when DON was metabolized by *Sphingomonas* sp. strain KSM1, involving three enzymes and NADH [95]. In this reaction, DON was transformed into 16-hydroxy-deoxynivalenol through a bacterial p450 catalytical system, which is related to the metabolism of toxins [104]. This system was also efficient against NIV and 3-ADON. This genus was also described as a DON degrader in a two-step reaction that generated 3-oxo-DON as intermediate and 3-epi-DON as the final product [94]. The strain used in this study, named SE-4, was isolated from wheat fields and was able to completely degrade the toxin in mineral media supplemented with 50 µg/mL at 28 °C.

Different degradation pathways between Gram-positive and Gram-negative bacteria were reported, as only Gram-positive bacteria were able to use DON as a carbon source, and they needed preincubation with the toxin to fully express this ability [82]. Both categories were able to form 3-epi-DON as an intermediate, so there are probably similarities in the degradation process performed by these bacteria, but further studies are necessary to clarify the degradation mechanisms suggested [82].

In recent years, continuous effort from many research groups has been dedicated to fully understanding the biodegradation mechanisms of deoxynivalenol and the role of each microorganism or enzyme in this process. Pioneer studies reported only on the degradation itself and subproducts, but recent work elucidated the entire enzymatic pathways, their cofactors, and optimal conditions for occurrence, such as temperature, pH, and oxygen presence or absence. However, complete studies are still scarce. The next steps may include expanding in-depth work to species already described as DON degraders to fully understand their degradation pathways. Then, another important issue to be figured out is how to deliver these active microorganisms, either in animal feed or in plant fields, as these environments may affect the microorganisms’ metabolism and, therefore, vomitoxin degradation.

### 2.5. Phylogenetic Analyses

Phylogenetic relationships provide an overview of evolutionary patterns between genes and species through phylogenetic trees, offering a structure to biological variation or traits.

We selected studies that provided microorganism identification and sample source and described which DON metabolite was produced in the biodegradation process (listed in Table 2). Studies that did not fit into these criteria were not considered when building the tree. 

The 16S rDNA gene is highly conserved among prokaryotes, not susceptible to horizontal exchange of genes, and the most used in bacterial taxonomic classification [105]. In this review, we used the 16S rDNA sequence provided by the authors of the articles gathered here to build a phylogenetic tree (Table S1), and similarities in DON metabolite production, particularly that of DOM-1, 3-keto-4-DON and 3-epi-DON, were sought. The 16S gene itself does not provide a means to assess the presence or activity of other genes involved in the production of these metabolites. Even so, some patterns were identified in the 16S phylogeny.

**Figure 4:**
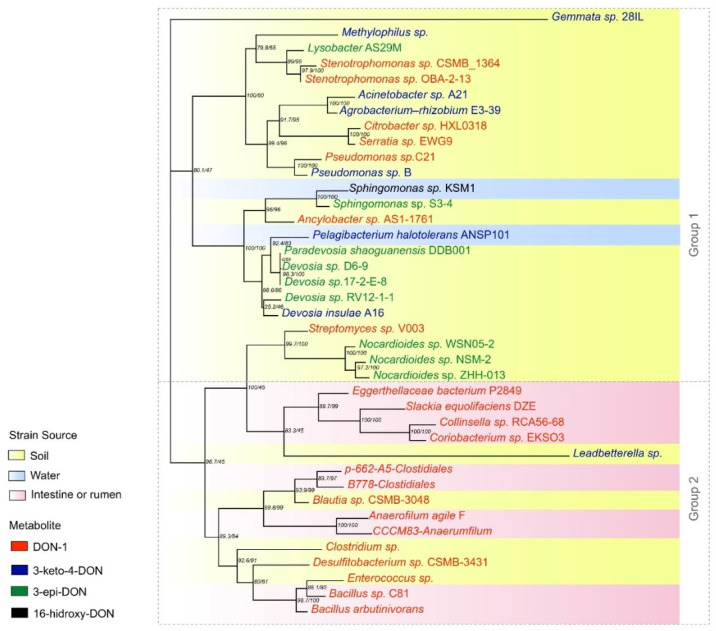
Phylogeny of 16S rRNA genes from organisms isolated from environmental or gastrointestinal sources that produce DOM-1, 3-keto-4-DON and 3-epi-DON metabolites.

The rRNA16S gene sequences of the strains were aligned using Clustal Omega [106] with default parameters. The inference of the maximum likelihood (ML) phylogenetic tree and the Bootstrap calculation were performed using the IQ-TREE software [107] using the ModelFinder method [108] for 1000 replicates. The tree figure (Figure 4) was organized using Adobe Illustrator CC 2017 software (version 21.0.0). In order to provide an overview of the evolutionary relationship of these organisms against a larger dataset, we also constructed a phylogenetic tree (Figure S1) using 205 16s rRNA (cited in the phylogenetic studies available in Table S2). Coherent distribution of these organisms was also observed.

Thirty-nine sequences fitting the criteria mentioned above were available. From these microorganisms, 32.5% were cultivated in anaerobiosis and 23% were also cultivated in aerobiosis. This information should be taken in account to define the application of these organisms, since agricultural practices involve aerobic environments. For animal application, these organisms may be used as feed supplements, as the GI tract is mostly anaerobic or microaerophilic.

Another important parameter to be considered for the final use of these organisms is the environmental pH where they would be applied. Studies tested pH levels ranging from 5 to 10 and observed the ability of microorganisms to degrade the toxin in these different pHs, not the survival of the microorganism. To be applied in animal systems, the microorganism must survive the acid pH of the stomach, but not necessarily keep their degradation activity in this pH, considering that the pH of the intestine is close to neutral. The intestine is the place where DON is mostly absorbed, especially in swine. Most studies gathered here used neutral pH (7.0). Eight articles used slightly acid pH (6.0), and another eight used alkaline pH (7.5 to 10).

Besides pH and atmospheric conditions, other facts must be considered, such as the microorganism’s capacity to proliferate and its ability to resist industrial processes such as drying or concentration. Bioprocessing itself may also involve some issues that decrease cell viability, e.g., shear stress [109]. The formation of spores may be an interesting characteristic to look for once it increases the microorganism’s resistance to thermal processes. From the studies gathered here, only two spore-forming organisms were reported, namely *Bacillus licheniformis* YB9 [75] and *Bacillus subtilis* ASAG 216 [76]. However, spore formation is not mandatory, as the only product approved for animal use with the ability to mitigate trichothecenes’ effects contains *Eubacterium* BBSH 797, a non-spore forming organism. Its recommended final dose is 1.7 × 10^8^ CFU/kg of feedstuff [110]. 

The main source of DON-degrading organisms described was either soil or plants, which together accounted for 70% of the isolated sequences (27). The rumen and intestine provided ten microorganisms able to degrade the toxin (25%). Only two strains were originated from water (5%), and one of these strains was also the only one registered to produce 16-hydroxy-deoxynivalenol. This indicates that soil and plants are most favorable sources to perform screening tests for DON-degrading microorganisms.

According to the distribution of branches on the tree, the formation of two distinct groups was observed: group 1, with mixed characteristics, with strains from different sources producing all three types of metabolites, but mainly 3-keto-DON and 3-epi-DON; and group 2, composed by strains from the intestine/rumen or soil producing DOM-1. The uncultured *Leadbetterella* sp. strain was not included in this group because of the low bootstrap. It is interesting to mention that those bacteria isolated from soil included in group 2, such as *Enterococcus* and *Blautia*, are commonly found in the intestine/rumen, showing a pattern of source/metabolite.

A random distribution was observed in group 1 regarding both source and metabolite produced. This can be explained by the multiple mechanisms described in the articles. Although only a few manuscripts described full metabolic pathways, including enzymes and cofactors involved in the process, some features - such as the active source of degradation (e.g., supernatant, cell lysate, or living cell) - demonstrated differences in the degradation process, indicating different evolutive pathways. 

Strains capable of producing 3-epi-DON as a DON metabolite were all soil-borne (*Nocardioides* sp. ZHH-013, *Nocardioides* sp. NSM-2, *Nocardioides* sp. WSN05-2, *Devosia* sp. 17-2-E -8, *Devosia* sp. RV12-1-1 and *Pseudomonas* sp. B). The same pattern was observed in strains that produced 3-keto-DON (uncultured *Leadbetterella* sp., *Devosia insulae* A16, *Agrobacterium* E3-39, *Acinetobacter* sp. A21, uncultured *Methylophilus*, and *Gemmata* sp. 28IL). Some articles reported 3-keto-DON as an intermediate in the enzymatic reaction leading to the formation of 3-epi-DON, which explains the similar pattern [86,102].

On the other hand, the origin of the strains that produce DOM-1 is variable. However, it was noted that 100% of the gut/rumen samples were composed of strains that produced this metabolite (*Bacillus* sp. C81, *Anaerophilum agile* F, *Coriobacterium* sp. EKSO3_Collinsella, and *Collinsella* sp. RCA56-68).

From the 21 articles gathered to build the tree, six reported production of DOM-1, five 3-keto-DON, and nine 3-epi-DON as the final metabolite of the DON degradation process. Because of the different cell lines used and methodologies applied in toxicology studies, it is not clear which of these three metabolites is least toxic. What is well known is that they are less toxic than DON itself. Therefore, biodegradation processes that form any of these by-products are useful to mitigate toxic effects [111].

## 3. Conclusions

Deoxynivalenol is proven to have different but deleterious toxic effects in both low and high doses in animal systems. Biodegradation is a very interesting approach to mitigate DON’s toxicity, even though most studies that isolated DON-degrading microorganisms still lacked in-depth descriptions of the metabolic pathways and the effects of the microorganism in vivo in food animals. Many genera were reported as DON degraders, ranging from several evolutive paths. A common root was observed between microorganisms that produced DOM-1 and their intestinal origin. Soil was the most common source used to find microorganisms with the ability to convert DON into less toxic compounds, and 3-epi-DON was the usual metabolite produced. Now, the physiology of theses microorganisms must be taken in account to develop effective products to mitigate the effects of this toxin in livestock production.

## Figures and Tables

**Table 1:** Recommended levels of deoxynivalenol in animal feed established by regulatory public agencies worldwide.

Agency	Specifications	Limit (mg/kg)	Reference
FDA (United States)	Grains and grain by-products destined for ruminating beef and feedlot cattle older than 4 months and for chicken	10	[14]
	Grain and grain by-products destined for swine and other animals	5	
EFSA (EU)	Cereals and cereal products except for maize by-products	8	[17]
	Maize by-products	12	
	Complementary and complete feeding stuffs for animals	5	
	Complementary and complete feeding stuffs for pigs	0.9	
Food Inspection Agency (Canada)	Diets for cattle and poultry	5	[15]
	Diets for swine, young calves, and lactating dairy animals	1	
Department of Agriculture, Forestry, and Fisheries (South Africa)	Feeding stuffs on a full ration basis for:		[16]
	Pigs	1	
	Cattle	5	
	Calves up to 4 months	2	
	Dairy Cattle	3	
	Poultry	4	

**Table 2:** Microorganisms described as deoxynivalenol degraders in vitro, their sources of isolation, the metabolites formed, and the conditions used in the relevant studies.

Microorganism	Source	Degradation Mechanism	Metabolite Formed	Cultivation Conditions	Degradation	References
*Acinetobacter*, *Leadbetterella*, and *Gemmata*	Soil	Not described	3-keto-4-DON	28 °C, 120 RPM	100 µg/mL, completely degraded after 7 days	[72]
*Agrobacterium–Rhizobium* strain E3-39	Soil	Oxidative reaction by extracellular enzyme	3-keto-4-DON	30 °C, vigorous shaking, pH 7	200 µg/mL, completely degraded within one day	[69]
*Eubacterium* sp. strain BBSH 797	Bovine rumen	Not described	DOM-1	37 °C, anaerobic	Completely degraded	[73,74]
*Bacillus licheniformis* YB9	Soil	Not described	Not described	37 °C	1 µg/mL, completely degraded in 2 days	[75]
*Bacillus subtilis* ASAG 216	Donkey intestine	Extracellular enzymes	Not described	35–45 °C, 200 RPM, pH 6.5–9.0	80% of 100 μg/mL within 8 h	[76]
*Bacillus* spp. LS-100 *Clostridiales, Anaerofilum* sp., *Collinsella* sp.	Chicken intestine	Not described	DOM-1	37 °C, anaerobic conditions	100 µg/mL, 80–100% degraded within 3 days	[77]
*Devosia mutans* 17-2-E-8	Alfalfa field soil	Intracellular enzymes (DepA and DepB)	3-keto-DON and 3-epi-DON	28 °C, 200 rpm, pH 6–8	100 µg/mL, 95% degraded within 2 days	[78,79,80,81]
*Devosia insulae* strain A16	Wheat field soil	Not described	3-keto-DON	35 °C, neutral pH	20 µg/mL, 88% degraded within 2 days	[60]
*Devosia* sp.	Soil	Intracellular enzymes	3-epi-DON	28 °C, 120 RPM	100 µg/mL, completely degraded within 1 day	[82]
*Devosia* sp. D6-9	Soil	Dehydrogenase and reductase	3-ketoDON and 3-epi-DON	28 °C, 220 rpm	500 µg/mL, completely degraded in 2 h	[83]
*Eggerthella* sp. DII-9	Chicken intestine	Not described	DOM-1	20–45 °C, pH 5–10, anaerobic	100 µg/mL, completely degraded within 3 days	[84]
*Lactobacillus rhamnosus* SHA113	Human milk	Not described	3-epi-DON	37 °C, pH 6	5 µg/mL, 60% degraded within 2 days	[85]
*Methylophilus, Ancylobacter, Pseudomonas*, and *Devosia* (bacteria consortium C20)	Soil	Not described	3-keto-DON	30 °C, pH 7	10 µg/mL, 74% degraded within 10 days	[86]
*Nocardioides* sp. strain WSN05-2	Wheat field soil	Not described	3-epi-DON	28 °C, agitation	1000 µg/mL, completely degraded after 10 days	[70]
*Nocardioides* sp. NSM2	Wheat leaves and soil	Not described	3-epi-DON	28 °C, 120 RPM	100 µg/mL, completely degraded within 1 day	[82]
*Nocardioides* sp. ZHH-013	Soil	Living cells	3-keto-DON and 3-epi-DON	30 °C, 220 RPM	33.747 mM, 80% degraded within 24 h	[87]
*Paradevosia shaoguanensis* DDB001	Wheat soil	Not described	3-epi-DON	30 °C, 220 RPM	200 µg/mL, completely degraded in 3 days	[88]
*Pelagibacterium halotolerans* ANSP101	Seawater	Intracellular protein	3-keto-DON	28 °C	50 µg/mL, 80% degraded after 12 h	[89]
*Pseudomonas, Clostridium*, and *Desulfitobacterium* (bacterial consortium PGC3)	Wheat field soil	Not described	DOM-1	20–37 °C, pH 5–10	100 µg/mL, completely degraded after 7 days	[90] [91]
*Pseudomonas* sp. Y1 and *Lysobacter* sp. S1 (bacteria consortium LZ-N1)	Soil samples	Enzymatic epimerization	3-epi-DON	30 °C, agitation	50 µg/mL, completely degraded within 2 days	[92]
*Serratia, Clostridium*, *Citrobacter*, *Enterococcus*, *Stenotrophomonas*, *Streptomyces*, and *Citrobacter freundii* A47	Soil	Not described	DOM-1	12–40 °C, pH 6.0–7.5, aerobic and anaerobic	50 µg/mL, 99% degraded within 60 h	[93]
*Sphingomonas* S3-4	Wheat fields	Oxidation mediated by aldo/keto reductase	3-keto-DON and 3-epi-DON	28 °C, 220 RPM	100 µg/mL, completely degraded in 3 days	[94]
*Sphingomonas* sp. strain KSM1	Lake water	P450 enzymes	16-hydroxy-DON	37 °C	300 µg/mL, completely degraded in 3 days	[95]
*Slackia* sp. D-G6	Chicken intestines	Not described	DOM-1	32–47 °C, pH 6–10, anaerobic	25 µg/mL, completely degraded in 1 day	[96]
*Stenotrophomonas* and *Blautia* (microbial consortium DX100)	Soil samples	De-epoxidation by intracellular reductases	DOM-1	20–40 °C, pH 6–7.5	50 µg/mL, 100% degraded in 2 days	[97]

## Data Availability

The data presented in this review paper are openly available in published papers listed in References.

## Supplementary Materials

The following supporting information can be downloaded at: https://www.mdpi.com/article/10.3390/toxins14020090/s1, Table S1. The 16S rRNA gene sequences of DON-degrading bacteria provided in each study, Table S2. The 16S rRNA gene sequences of DON-degrading bacteria and other bacteria used in each phylogenetic study provided in the articles, Figure S1: The 16S rRNA genes from 205 organisms listed in Table S2. Highlighted (red) are the 40 organisms shown in Figure 1 and Table S1. References [111,112,113,114,115] are cited in the supplementary materials.
