# Supplementary material for: Deoxynivalenol: Toxicology, Degradation by Bacteria, and Phylogenetic Analysis
Source: Toxins (Basel). 2022 Jan 25;14(2):90. doi: 10.3390/toxins14020090 (PMC8876347; doi:10.3390/toxins14020090)
Supplement: Supplementary file 1 [file toxins-14-00090-s001.zip › toxins-1541315-supplementary.pdf]

Deoxynivalenol: Toxicology, Degradation by Bacteria, and Phylogenetic Analysis

Anne Caroline Schoch Marques Pinto, Camilla Reginatto De Pierri, Alberto Gonçalves Evangelista, Ana Silva de Lara Pires Batista Gomes and Fernando Bittencourt Luciano

Table S1. 16S rRNA gene sequences of DON degrading bacteria provided in each study.

Organism	Strain	Names used in Figure 4	Accession Number	Study
Acinetobacter sp.	A21	*Acinetobacter sp.* A21	AM179861.1	[72]
Bacterium sp.	E3-39	*Agrobacterium-rhizobium* E3-39	KJ680277.1	[69]
Anaerofilum agile	F	*Anaerofilum agile* F	NR_029315.1	[77]
Ancylobacter sp.	AS1.1761	*Ancylobacter sp.* AS1.1761	AY056830.1	[86]
Bacillus arbutinivorans	-	*Bacillus arbutinivorans*	AF519469.1	[77]
Bacillus sp.	C81	*Bacillus sp.* C81	AM179887.1	[77][112]
Citrobacter sp.	HXL0318	*Citrobacter sp.* HXL0318	MN419325.1	Unpublished*
Collinsella sp.	RCA56-68	*Collinsella sp.* RCA56-68	AB031063.1	[77]
Coriobacterium sp.	EKSO3	*Coriobacterium sp.* EKSO3	AJ245921.1	[77]
Costridium sp.	-	*Clostridium sp.*	L23477.1	[113]*
Devosia insulae	A16	*Devosia insulae* A16	EU794908.1	[60]
Devosia sp	D6-9	*Devosia sp.* D6-9	NZ_CP045919.1	[83]
Devosia sp.	17-2-E-8	*Devosia sp.*17-2-E-8	KJ572863.1	[78][79]
Devosia sp.	RV12-1-1	*Devosia sp.* RV12-1-1	MT520548.1	[82]
Eggerthellaceae bacterium Marseille	P2849	*Eggerthellaceae bacterium* P2849	LT576395.1	[84]
Enterococcus sp.	-	*Enterococcus sp.*	AJ132470.1	[114]*
Gemmata sp.	28IL	*Gemmata sp.*28IL	KC169940.1	[72]
Lysobacter sp.	S1	*Lysobacter sp.* S1	MN197751	[92]
Nocardioides sp	ZHH-013	*Nocardioides sp.* ZHH-013	MW493343.1	[87]
Nocardioides sp.	NSM-2	*Nocardioides sp.* NSM-2	MZ436976.1	[82]
Nocardioides sp.	WSN05-2	*Nocardioides sp.* WSN05-2	AB282680.1	[70]
Paradevosia shaoguanensis	DDB001	*Paradevosia shaoguanensis DDB001*	NZ_CP068983.1	[88]
Pelagibacterium halotolerans	ANSP101	*Pelagibacterium halotolerans* ANSP101	MN795607.1	[89]
Pseudomonas sp.	C21	*Pseudomonas sp.* C21	AM179883.1	[90]
Pseudomonas sp.	Y1	*Pseudomonas sp.* Y1	MN197750	[92]
Serratia sp.	EWG9	*Serratia sp.* EWG9	MW205826.1	Unpublished*
Slackia equolifaciens	DZE	*Slackia equolifaciens DZE*	NR_116295.1	[96]
Sphingomonas sp.	KSM1	*Sphingomonas sp.* KSM1	AB744218.1	[95]
Sphingomonas sp.	S3-4	*Sphingomonas sp.* S3-4	KY575150.1	[94]
Stenotrophomonas sp.	OBA 2.13	*Stenotrophomonas sp.* OBA-2-13	OK039227.1	[97]
Streptomyces sp.	V003	*Streptomyces sp.* V003	MH298059.1	Unpublished*
Uncultured bacterium clone B778	B778	*B778 Clostidiales*	AY984815.1	[77]
Uncultured bacterium clone p-662-A5	p-662-A5	*p-662-A5 Clostidiales*	AF371567.1	[77]
Uncultured Blautia sp.	CSMB_3048	*Blautia sp.* CSMB-3048	MF896357.1	[97]
Uncultured Desulfitobacterium	CSMB_3431	*Desulfitobacterium sp.* CSMB-3431	MF896716.1	[90]
Uncultured Leadbetterella sp.	OTU2305	*Leadbetterella sp.*	LT858987.1	[72]
Uncultured Methylophilus sp.	SV424	*Methylophilus sp.*	JQ858779.1	[86]
Uncultured Stenotrophomonas	CSMB_1364	*Stenotrophomonas sp.* CSMB_1364	MF894792.1	[23]
Unidentified bacterium clone CCCM83	CCCM83	*CCCM83-Anaerumfilum*	AY654968.1	[77]

*These genera were used in the study by Islam and colleagues [24]

Table S2. 16S rRNA gene sequences of DON degrading bacteria and other bacteria used in each phylogenetic study provided in the articles.

Organisms	Strain	Accession Number	Characterized or cited by
Acinetobacter sp.	*A21*	*AM179861.1*	[72]
Achromobacter marplatensis	R-46660	NR_117614	
Burkholderia multivorans	Struelens	NR_029358.1	
Gemmata sp.	28IL	KC169940.1	
Uncultured Leadbetterella sp.	OUT2305	LT858987.1	
Bacterium sp.	E3-39	KJ680277.1	[69]
Pseudomonas aeruginosa	-	X06684.1	
Pseudomonas andropogonis	-	X67037.1	
Bacillus subtilis	-	X00007	
Escherichia coli	-	J01859.1	
Pseudomonas caryophylli	-	X67039.1	
Acetobacter aceti	-	X74066.1	
Azospirillum lipoferum	Ncimb11861	Z29619.1	
Sphingomonas adhaesiva	-	D16146	
Bradyrhizobium japonicum	-	U69638.3	
Blastobacter denitrificans	LMG 8443	S46917	
Rhodopseudomonas pseudopalustris	DSM 123	L11664.1	
Methylobacterium extorquens	JCM 2802	D32224	
Agrobacterium sp.	-	AB006037	
Agrobacterium rubi	LMG 156	X67228.1	
Agrobacterium tumefaciens	-	D14500.1	
Agrobacterium vitis	LMG 8750	X67225.2	
Rhizobium galegae	LMG 6214	X67226.2	
Rhizobium tropici	LMG 9518	X67233.1	
Mesorhizobium loti	LMG 6125	X67229.2	
Rhizobium tropici	IAM 14206	D12798.1	
Agrobacterium rhizogenes	IAM 13570	D12788.1	
Mesorhizobium huakuii	IFO 15243	D13431.1	
Anaerofilum agile	F	NR_029315.1	[77]
Bacillus arbutinivorans	-	AF519469.1	
Bacillus sp.	C81	AM179887.1	
Collinsella sp.	RCA56-68	AB031063.1	
Coriobacterium sp.	EKSO3	AJ245921.1	
Uncultured bacterium clone B778	B778	AY984815.1	
Uncultured bacterium clone p-662-A5	p-662-A5	AF371567.1	
Unidentified bacterium clone CCCM83	CCCM83	AY654968.1	
Ancylobacter sp.	AS1.1761	AY056830.1	[86]
Uncultured Methylophilus sp.	SV424	JQ858779.1	
Hyphomicrobium nitrativorans	NL23	NR_121713.2	
Acidovorax wautersii	NF 1078	NR_118410.1	
Prosthecomicrobium hirschii	16	NR_104906.1	
Taonella mepensis	H1	NR_132292.1	
Bosea thiooxidans	BI-42	NR_114668	
Terrimonas soli	FL-8	NR_159891	
Citrobacter sp.	HXL0318	MN419325.1	Unpublished
Costridium sp.	-	L23477.1	[113]
Devosia insulae	A16	EU794908.1	[60]
Devosia sp	D6-9	NZ_CP045919.1	[83]

Devosia sp.	17-2-E-8	KJ572863.1	[79]
Devosia soli	GH2-10	DQ303125.1	
Devosia crocina	IPL20	EF433461.1	
Devosia riboflavina	DSM 7230	AJ549086.1	
Devosia subaequoris	HST3-14	AM293857.1	
Devosia lucknowensis	L15	JN687580.1	
Devosia chinhatensis	IPL18	EF433462.1	
Devosia epidermidihirudinis	E84T	KC254735.1	
Devosia sp	R-21940T	AJ786801.1	
Devosia submarina	Sl74	AB712348.1	
Devosia psychrophila	Cr7-05	GU441678.1	
Devosia glacialis	Cr4-44	HM474794.1	
Devosia yakushimensis	-	AB361068.1	
Devosia neptuniae	J1	AF469072.1	
Devosia insulae	DS-56	EF012357.1	
Devosia humi	-	KM598259.1	
Prosthecomicrobium enhydrum	9b	GQ221761.1	
Prosthecomicrobium mishustinii	17	FJ560749.1	
Devosia pacifica	NH131	KF111722.1	
Devosia geojensis	BD-c194	EF575560.1	
Devosia sp.	BBB001	JX392051.1	
Devosia albogilva	IPL15	EF433460.1	
Devosia honganensis	NSL10	KP339871.1	
Youhaiella tibetensis	fig4	KF740588.1	
Paradevosia shaoguanensis	J5-3	KC222641.1	
Pelagibacterium luteolum	1_C16_27	EF540455.1	
Pelagibacterium halotolerans	B2	EU709017.1	
Cucumibacter marinus	DSM 18995	EF211830.2	
Zhangella mobilis	E6	EU255260.1	
Maritalea myrionectae	CL-SK30	EF988631.1	
Carbophilus carboxidus	CIP 105722	JN175336.1	
Aminobacter aminovorans	DSM7048T,	AJ011759.1	
Pseudaminobacter salicylatoxidans	-	AF072542.1	
Nitratireductor aquibiodomus	NL21	AF534573.1	
Nitratireductor pacificus	pht-3B	DQ659453.1	
Blastochloris viridis	-	D25314.1	
Rhodoplanes roseus	-	D25313.2	
E.coli	ATCC 11775T	X80725.1	
Devosia sp.	RV12-1-1	MT520548.1	[82]
Nocardioides sp.	NSM-2	MZ436976.1	
Eggerthellaceae bacterium Marseille	P2849	LT576395.1	[84]
Gordonibacter urolithinfaciens	AA00211	LT223667.1	
Gordonibacter urolithinfaciens	CEBAS 1/15P	NR_134044.1	
Gordonibacter faecihominis	CAT-2	KF785806.1	
Gordonibacter pamelaeae	JCM 16334	NR_113189.1	
Gordonibacter sp. Marseille-P2775	P2775	LT558845.1	
Eggerthella sp.	YY7918	AB379693.1	
Gordonibacter sp. Marseille-P3078	P3078	LT598545.1	
Eggerthella sinensis	JCM14551	NR_042840.1	
Eggerthella timonensis	P3135T	LT598568.1	
Eggerthella lenta	AUH-Julong365	JN874873.1	
Eggerthella lenta	CAT-1	JF798636.1	
Eggerthella lenta	1899B	NR_037089.1	

Eggerthella lenta	JCM 9979	NR_113158.1	
Eggerthellaceae bacterium Marseille-P2849	P2849	LT576395.1	
Escherichia coli	44A	KP789331.1	
Enterococcus sp.	-	AJ132470.1	[114]
Lysobacter sp.	S1	MN197751	
Pseudomonas sp.	Y1	MN197750	
Pseudomonas sp.	PVR-YHB-1-2	KP986946.1	
Pseudomonas sp	CoA5	Ky094373	
Pseudomonas resinovorans	SS1	KP453781.1	
Pseudomonas sp.	CMF-14	MG266389.1	
Pseudomonas sp.	MI-45a	DQ180955.1	
Pseudomonas sp.	XT-29	KR063557.1	
Pseudomonas sp.	HI-G1	DQ205300.1	
Pseudomonas aeruginosa	PP-10	KF186662.1	
Pseudomonas sp	BPS-8	C273520.1	
Pseudomonas sp.	J11	AB097174.1	
Pseudomonas resinovorans	LAM 9	EU019983.1	
Pseudomonas psudoalcaligenes	X4	KJ586277.1	[92]
Pseudomonas alcaligenes	-	FJ455454.1	
Pseudomonas sp.	C2-AL	LN913074.1	
Lysobacter sp.	MHS03	DQ993327.1	
Lysobacter sp.	Sq12	KX255010.1	
Lysobacter enzymogenes	SEMP3	JX915820.1	
Lysobacter enzymogenes	BB14	FJ657670.1	
lysobacter gummosus	CP72	KF040972.1	
Lysobacter sp.	DC2b-58	AB552868.1	
Lysobacter sp.	R5-394	JQ659762.1	
Lysobacter niastensis	TSH57	MG600267.1	
Lysobacter panacisoli	SR-15	KX082844.1	
Lysobacter sp.	GK19	MK424386.1	
Lysobacter sp.	QT22	GU385868.1	
Nocardioides sp	ZHH-013	MW493343.1	
Nocardioides sp.	THG-DN5.4	KM035951.2	
Nocardioides nitrophenolicus	NSP 41	AF005024.1	
Nocardioides kongjuensis	A2-4	DQ218275.1	
Nocardioides albidus	-	KM073954.1	
Nocardioides sp.	MN8	FJ423551.1	[87]
Nocardioides vastitatis	-	MK787305.1	
Nocardioides pelophilus	THG-T63	KY287247.1	
Nocardioides sp.	LS1	AB627759.1	
Escherichia coli	NBRC 102203	AB681728.1	
Nocardioides sp.	WSN05-2	AB282680.1	
Nocardioides aquiterrae	GW-9	AF529063.1	
Nocardioides sp.	OS4	U61298.1	
Nocardioides kribbensis	KSL-2	AY835924.1	
Nocardioides aestuari	JC2056	AY423719.2	
Nocardioides sp.	AN3	AB183711.2	
Nocardioides plantarum	NCIMB 12834	AF005008.1	[70]
Nocardioides kongjuensis	A2-4	DQ218275.1	
Nocardioides panaciterrae	-	AB257719.1	
Nocardioides alkalitolerans	KSL-1	AY633969.1	
Nocardioides dubius	-	AY928902.1	
Nocardioides jensenii	-	AF005006.1	

Streptomyces coelicolor	-	X60514.1	
Paradevosia shaoguanensis	DDB001	NZ_CP068983.1	[88]
Pelagibacterium halotolerans	ANSP101	MN795607.1	
Pelagibacterium halotolerans	RB 47	KJ939460.1	
Devosia riboflavina	IFO 13584	NR_115565.1	[89]
Ancalomicrobium adetum	NBRC 102456	NR_104726.1	
Methylorhabdus multivorans	DM13	NR_024844.1	
Pseudomonas sp.	C21	AM179883.1	[90]
Uncultured Desulfitobacterium	CSMB_3431	MF896716.1	
Serratia sp.	EWG9	MW205826.1	Unpublished
Slackia equolifaciens	DZE	NR_116295.1	
Slackia faecicanis	5WC12	NR_042220.1	
Slackia piriformis	YIT 12062	NR_113272.1	
Slackia exigua	JCM 11022	LC007113.1	
Slackia sp. NATTS	*NATTS*	AB505075.1	
Adlercreutzia sp. Marseille-P7992	-	LR031293.1	
Enterorhabdus sp.	Z73	MK287693.1	
Enterorhabdus muris	D2-1X-25	MK287668.1	[96]
Adlercreutzia equolifaciens	FJC-D53	AB306663.1	
Asaccharobacter celatus	JCM 14811	NR_114402	
Paraeggerthella hongkongensis	W04014	KP944194.1	
Eggerthellaceae bacterium	AT8	LN881601.1	
Atopobium parvulum	-	AF292372	
Raoultibacter massiliensis	P2849	NR_144751.1	
Sphingomonas sp.	KSM1	AB744218.1	
Sphingomonas panni	C52	AJ575818.2	
Sphingomonas mucosissima	CP173-2	AM229669.1	
Sphingomonas sp. JSS-26	JSS-26	AF131296.1	
Sphingomonas changbaiensis	V2M44	EU682685.1	
Sphingomonas pituitosa	EDIV	AJ243751.1	
Sphingomonas sanxanigenens	NX02	DQ789172.1	
Sphingomonas wittichii	RW1	NR_027525.1	
Sphingomonas fennica	K101	NR_041948.1	
Sphingobium yanoikuyae	-	D13728.1	
Sphingobium sp. SYK-6	SYK-6	NC_015976.1	[95]
Sphingomonas xenophaga	BN6	X94098.1	
Sphingomonas sp. YT	YT	AB047364.1	
Sphingobium rhizovicinum	CC-FH12-1	EF465534.1	
Novosphingobium aromaticivorans	DSM 12444	NR_074261.1	
Novosphingobium subterraneum	-	AB025014.1	
Novosphingobium panipatense	SM16	EF424402.1	
Sphingopyxis ginsengisoli	Gsoil 250	AB245343.1	
Sphingopyxis witflariensis	-	AJ416410.1	
Sphingobium japonicum	UT26S	NR_102886.2	
Sphingomonas sp.	S3-4	KY575150.1	[94]
Stenotrophomonas sp.	OBA 2.13	OK039227.1	
Streptomyces sp.	V003	MH298059.1	
Uncultured Blautia sp.	CSMB_3048	MF896357.1	[97]
Uncultured Stenotrophomonas	CSMB_1364	MF894792.1	
Streptomyces alkaliphilus	No. 7	NR_136864	
Citrobacter freundii	A47	LNFS00000000.1	[115]

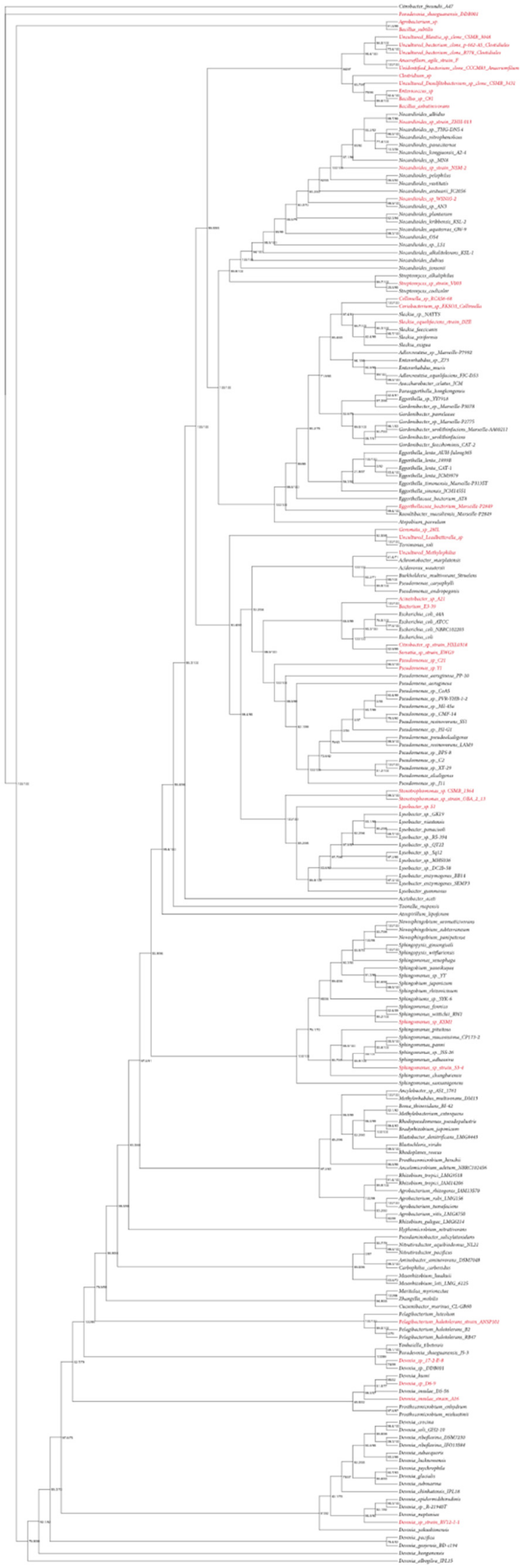

Figure S1. 16S rRNA genes from 205 organisms listed in Table S2. Highlighted (red) are the 39 organisms shown in Figure 4 and Table S1
